# Proposal for standardizing normal insulin ranges in Brazilian patients and a new classification of metabolic syndrome

**DOI:** 10.3389/fmed.2022.984001

**Published:** 2022-09-09

**Authors:** Pedro Renato Chocair, Precil Diego Miranda de Menezes Neves, Victor Augusto Hamamoto Sato, Sara Mohrbacher, Érico Souza Oliveira, Leonardo Victor Barbosa Pereira, Alessandra Martins Bales, Fagner Pereira da Silva, John A. Duley, Américo Lourenço Cuvello-Neto

**Affiliations:** ^1^Internal Medicine and Nephrology Service, Hospital Alemão Oswaldo Cruz, São Paulo, Brazil; ^2^Nursing Department, Hospital Alemão Oswaldo Cruz, São Paulo, Brazil; ^3^School of Pharmacy, The University of Queensland, Brisbane, QLD, Australia

**Keywords:** diabetes, HOMA-IR, insulin, hyperinsulinemia, obesity, metabolic syndrome

## Abstract

**Background:**

Insulin resistance and/or hyperinsulinemia are closely linked to adiposity, metabolic syndrome (MetS) and prolonged inflammatory processes.

**Methods:**

We retrospectively analyzed 1,018 adult individuals with a mean age of 46 years (74% male) and classified them as: Metabolically normal: without any of the five criteria of the International Diabetes Federation (IDF) used for the diagnosis of MetS, plus normal fasting insulin (Men < 8 mU/L, Women < 10 mU/L); Level 1 MetS: with one or two IDF criteria, plus hyperinsulinemia (Men: ≥ 8 mU/L), and Women: ≥ 10 mU/L); Level 2 MetS: with three or more IDF criteria, plus hyperinsulinemia.

**Results:**

The mean values for fasting insulinemia in metabolically normal individuals was 4.6 ± 1.8 mU/L and 5.6 ± 2.3 mU/L, while their means for the Homeostatic Model Assessment for Insulin Resistance (HOMA-IR) were 1.0 and 1.2 for men and women, respectively. In addition, the mean values for insulin (and HOMA-IR) for individuals with two normal anthropometric parameters (body mass index and waist girth), or two normal anthropometric parameters plus no IDF criteria, were similar to the metabolically normal group. Based on the obtained mean + 2 SD, we established the following insulin (and HOMA-IR) values as diagnostic cut-offs for hyperinsulinemia: Men: ≥ 8 mU/L (≥ 1.5), and Women: ≥ 10 mU/L (≥ 2.0). The mean serum insulin was significantly higher for individuals with Level 1 MetS (approx. 9 mU/L for both genders) compared with metabolically normal individuals, as was the prevalence of hepatic steatosis, which was more evident in men. Thus, the presence of one or two abnormal IDF criteria, combined with hyperinsulinemia and/or raised HOMA-IR, suggests the presence of MetS and insulin resistance. Patients of both genders with Level 2 MetS had higher serum insulin and/or HOMA-IR values than Level 1, as well as a higher prevalence of hypertension and hepatic steatosis, being more pronounced among men. The process was progressive and proportional to the degree of hyperinsulinemia.

**Conclusion:**

It is proposed that intervention against MetS progression should be started in individuals with Level 1 MetS, rather than waiting for more criteria for diagnostic confirmation, which this should help to reduce the occurrence of known complications such as type 2 diabetes, atherosclerosis, hypertension, and chronic kidney disease, among others.

## Background

Metabolic syndrome (MetS) is a serious disorder that results from prolonged subclinical systemic inflammation originating in adipose tissue (1–4). It is increasingly prevalent worldwide (5, 6) and threatens the continued growth of life expectancy that has been observed over the last two centuries (7).

The major factor responsible for its increased prevalence is certainly the pandemic of obesity, which is primarily caused by diets containing an excess of carbohydrates and by sedentary lifestyles (1, 8, 9). Yet, despite its recognized importance, there is no homogeneous definition of MetS. The most commonly used definitions are those of the European Group for the Study of Insulin Resistance, (10) the American Association of Clinical Endocrinologists, (11) the International Diabetes Federation (IDF), (12) and the National Cholesterol Education Program Adult Treatment Panel III 2005 (13). The first three consider insulin resistance to be a mandatory condition for diagnosis, while the latter requires the presence of three or more of the following five IDF criteria: (1) triglycerides ≥ 150 mg/dL; (2) high density lipoprotein-cholesterol (HDL-C) < 40 mg/dL in men and < 50 mg/dL in women, or patient receiving fibrate; (3) blood glucose ≥ 100 mg/dL; (4) increased waist girth (variable with ethnicity); and (5) hypertension ≥ 130 and/or/85 mm Hg, or patient receiving antihypertensive treatment (13).

Insulin resistance, hyperinsulinemia, and MetS are closely related to each other and to various medical complications such as type 2 diabetes, chronic kidney disease, hepatic steatosis, neoplasms, urinary calculosis, polycystic ovary syndrome, arthropathies, hypertension, cardiovascular diseases, and skin diseases including psoriasis (13–24).

Despite the recognized importance of MetS, the limits for diagnosing hyperinsulinemia and insulin resistance are still not clear. In this paper, we aimed to define normal values for insulin and the Homeostatic Model Assessment for Insulin Resistance (HOMA-IR) index based on a Brazilian cohort, and to suggest a new and simpler classification for MetS. We propose that this will provide early recognition and promote preventive therapeutic intervention for complications.

## Materials and methods

### Participants and study design

This was a cross-sectional, retrospective, single-center study that aimed to define the diagnostic cut-offs for fasting blood insulin and HOMA-IR in Brazilian adults of both genders. A cohort of 1,015 adult study participants (751 men, 264 women), with a mean age of 46.5 years ± 8.91, were recruited from participants who underwent elective screening consultations (check-ups) from October 2020 to March 2021. Patients were examined for comorbidities such as hypertension, diabetes, dyslipidemia, and obesity, due to their relevance to the proposed analyses.

Patient numbers varied slightly in different categories of results as some participants did not undergo a specific examination (i.e., ultrasound), and these have been noted in the text.

This study was approved by the Ethics and Research Committee of Hospital Alemão Oswaldo Cruz de São Paulo (CAAE: 46489021.6.0000.0070).

### Clinical evaluation and sample collection for laboratory tests

Blood pressure was measured during clinical evaluations using the auscultatory method with a manual sphygmomanometer, in a seated position after at least 5 min of rest. Waist girth (cm) was measured at the midpoint between the last rib and the iliac crest. Body mass index (BMI) was calculated after confirming weight and height.

The five criteria used by the IDF for the diagnosis of MetS (13)—as listed above—were assessed, and each participant also authorized an additional blood sample and an isolated urine sample to be used for the determination of fasting serum insulin and microalbuminuria. The samples were collected after 10–12 h fasting and were analyzed in addition to the standard tests pre-defined by the check-up program of Hospital Alemão Oswaldo Cruz (São Paulo), at no additional cost to customers, companies, or healthcare providers.

Patients were considered normal if they had no previous diagnosis of the conditions considered criteria for MetS and which were not on regular use of drugs to treat them. Patients were diagnosed with MetS if they fulfilled the diagnostic criteria according to IDF criteria.

The standard laboratory analyses were conducted on the day of the allocated appointment, with their respective evaluation methods and reference values considered by the analysis laboratory, and included:

• Creatinine, kinetic method, colorimetric
• Blood glucose: enzymatic method
• Fasting insulin: electro chemiluminometric assay
• HOMA-IR (Homeostatic Model Assessment for Insulin Resistance
• HDL-C (high density lipoprotein cholesterol): homogeneous enzyme assay
• Triglycerides: enzymatic assay
• Microalbumin in an isolated urine sample: by immunoturbidimetry (mg/g creatinine).

In addition, hepatic steatosis was assessed using abdominal ultrasound by the same team and equipment.

### Statistical analysis

The D’Agostino-Pearson omnibus and Shapiro–Wilk tests were used to assess the distribution of variables. Variables with parametric distribution were expressed as mean ± standard deviation and compared using the Student’s *t*-test when in two groups and using analysis of variance (ANOVA) when in three or more groups. Where data distributions were non-parametric, the variables were expressed as median and interquartile ranges, and compared using the Mann–Whitney *U* test for two groups or the Kruskal–Wallis test among three or more groups. Nominal variables were expressed as absolute and percentage counts and were analyzed using the Chi-squared or Fisher’s exact test. We used the analysis of Receiver Operator Characteristics (ROC) curves to evaluate the accuracy of insulin and HOMA-IR values for the prediction of metabolic syndrome for male and female patients. Values of *p* < 0.05 or, in the case of multiple comparisons, *q* less than 0.05 were considered statistically significant. Statistical analyses were performed using GraphPad Prism version 8.00 (GraphPad Software, San Diego, CA, United States) and SPSS 25.0 (IBM Corp, Armonk, NY, United States) software programs.

## Results

The cohort of 1,015 adult study participants (751 men, 264 women) had a mean age of 46.5 ± 8.9 years old. The baseline characteristics of patients are described on Table 1. The relationship between ranges of two anthropometric parameters (BMI, Waist girth) compared with insulin and HOMA-IR are described on Table 2.

**TABLE 1:** Baseline characteristics of patients.

Variables	Men (*n* = 751)	Women (*n* = 264)	Total (*n* = 1015)
Age (years)	47.3 ± 9.7	44.8 ± 7.1	46.5 ± 8.9
Body Mass Index (kg/m^2^)	28.2 ± 3.8	25.4 ± 4.17	27.4 ± 4.1
Waist Girth (cm)	100.6 ± 10.9	87.6 ± 10.9	96.6 ± 12.4
Systolic blood pressure (mmHg)	127.3 ± 13.5	117.9 ± 15.7	124.5 ± 14.9
Diastolic blood pressure (mmHg)	85.1 ± 9.9	75.2 ± 11.1	82.1 ± 11.3
Total Cholesterol (mg/dL)	187.8 ± 35.9	186.1 ± 36.2	187.3 ± 36.0
LDL (mg/dL)	124.0 ± 72.9	109.6 ± 32.6	119.6 ± 63.6
HDL (mg/dL)	47.7 ± 12.9	60.4 ± 15.9	51.6 ± 15.1
Triglycerides (mg/dL)	124.5 ± 70.4	92.2 ± 50.2	114.7 ± 66.5
Fasting glucose (mg/dL)	96.2 ± 13.7	91.2 ± 8.4	94.7 ± 12.5
Insulin (mU/L)	12.1 ± 8.4	9.9 ± 6.2	11.4 ± 7.8
HOMA-IR	2.9 ± 2.3	2.3 ± 1.53	2.71 ± 2.0
HbA1C (%)	5.3 ± 0.5	5.2 ± 0.5	5.28 ± 0.5
Hepatic Steatosis (n/%)	299/743 (40.2)	43/255 (16.9)	342/998 (33.7)
Serum creatinine (mg/dL)	1.8 ± 0.6	0.7 ± 0.2	1.46 ± 7.6

**TABLE 2:** Anthropometric parameters of BMI and Waist Girth compared to insulin and HOMA-IR in men and women.

BMI ranges	n (%)	Insulin (mU/L) mean ± SD	HOMA-IR mean ± SD
**Men**			
17.8–22.9	51 (6.7%)	5.80 ± 2.88^a^**	1.34 ± 0.69^b^**
23–24.9	121 (16.1%)	7.52 ± 4.91^a^**	1.77 ± 1.18 ^b^**
25–29.9	374 (49.6%)	10.4 ± 5.58 ^a^**	2.46 ± 1.40 ^b^**
≥ 30	207 (27.5%)	17.2 ± 14.3 ^a^**	4.26 ± 3.92 ^b^**
**Women**			
17.8–22.9	91 (34.3%)	6.35 ± 2.50^c^**	1.38 ± 0.60^d^*
23–24.9	45 (17%)	7.93 ± 3.17^c^**	1.76 ± 0.69^d^*
25–29.9	88 (33.2%)	10.6 ± 5.83^c^**	2.46 ± 1.48^d^*
≥ 30	41 (15.4%)	15.4 ± 7.23^c^**	3.73 ± 2.02^d^*

**Waist ranges (cm)**	**n (%)**	**Insulin (mU/L)** **mean ± SD**	**HOMA-IR** **mean ± SD**

**Men**			
< 90	106 (14.1%)	6.30 ± 4.00^e^**	1.47 ± 0.98^f^*
90–99.9	266 (35.4%)	9.43 ± 5.53^e^**	2.21 ± 1.35^f^*
≥ 100	380 (50.5%)	14.4 ± 11.5^e^**	3.52 ± 3.14^f^*
**Women**			
< 80	64 (24%)	6.05 ± 2.58^g^**	1.31 ± 0.58^h^**
80–89.9	98 (37%)	7.87 ± 3.30^g^**	1.76 ± 0.83^h^**
≥ 90	102 (38.6%)	13.0 ± 6.89^g^**	3.08 ± 1.83^h^**

a-h: comparison among groups according to the respective letter. For BMI (753 men, 265 women, 1,018 total), insulin and HOMA-IR means were significantly different by *p < 0.05 or **p < 0.005; for waist girth (752 men; 264 women; waist not recorded for 1 man and 1 woman; 1,016 total), means differed by *p < 0.05 or **p < 0.001.

The lowest ranges of these two anthropometric parameters were used to define normal insulin and HOMA-IR values: BMI < 23 for men and women; Waist < 90 cm and < 80 cm for men and women, respectively. These anthropometric normal ranges were then combined in an analysis with the five IDF criteria for MetS (Table 2).

Based on the mean + 2 SD values for insulin and HOMA-IR observed in Table 3, as well as a ROC curve co-ordinates, we defined the following diagnostic cut-offs for hyperinsulinemia (and raised HOMA-IR): Men ≥ 8 mU/L (sensitivity: 82.4% and specificity: 58%) and HOMA-IR ≥ 1.5 (sensitivity: 91.3% and specificity: 51%) and Women ≥ 10 mU/L (sensitivity: 78.6% and specificity: 72.5%) and HOMA-IR ≥ 2.0 (sensitivity: 83.9% and specificity: 70.2%).

**TABLE 3:** Insulin and HOMA-IR values of participants with: (**A**) normal anthropometric parameters (BMI and Waist); (**B**) no IDF criteria; or **(C)** normal anthropometric parameters (BMI and Waist) and no IDF criteria.

Gender		Insulin (mU/L)				HOMA-IR			
			
	n (%)	Mean	SD	Mean + 1 SD	Mean + 2 SD	Mean	SD	Mean + 1 SD	Mean + 2 SD
**(A)**									
Men	18 (2.4%)	4.3^a^*	1.4	5.7	7.1	1.0^d^*	0.35	1.35	1.70
Women	38 (14%)	5.4^a^*	2.3	7.7	10	1.2^d^*	0.5	1.7	2.20
**(B)**									
Men	49 (6.5%)	4.6^b^*	1.8	6.4	8.2	1.0^e^*	0.4	1.4	1.8
Women	48 (18%)	5.6^b^*	2.3	7.9	10.2	1.2^e^*	0.5	1.7	2.2
**(C)**									
Men	11 (1.5%)	4.0^c^*	1.6	5.6	7.2	0.9^f^*	0.3	1.2	1.5
Women	33 (4.4%)	5.3^c^*	2.2	7.5	9.7	1.1^f^*	0.5	1.6	2.1

Normal anthropometric ranges were defined as: BMI < 23; Waist < 90 (men), < 80 (women); a-f: comparison among groups according to the respective letter. *Insulinemia and HOMA-IR means were statistically different between men and women (*p* < 0.005). n = 752 men (1 man excluded as insulin not recorded); 265 women; 1,017 total.

Based on those data, a new classification for diagnosis of MetS is proposed:

• Metabolically normal: fasting insulinemia Men < 8 mU/L, Women < 10 mU/L, and no IDF criteria for the diagnosis of MetS.
• Level 1 MetS: hyperinsulinemia, plus one or two IDF criteria for the diagnosis of MetS.
• Level 2 MetS: hyperinsulinemia, plus three or more IDF criteria for the diagnosis of MetS.

**FIGURE 1:**
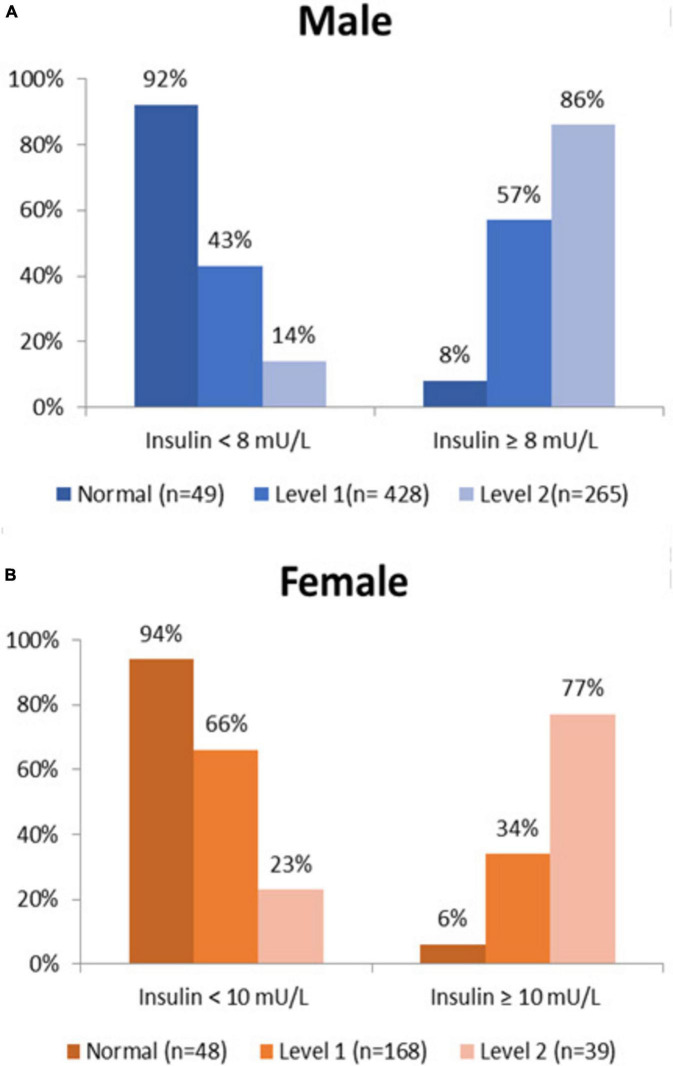
Proportion of normal and MetS patients vs. insulin cut-offs for **(A)** men and **(B)** women.

The proportion of metabolically normal and MetS patients (Level 1 and Level 2) according to insulinemia below and above the established cut-off may be observed in Figure 1. The statistical differences for the men with insulinemia ≥ 8 mU/L were highly significant (*p* < 0.001) between normal, Level 1 MetS, and Level 2 MetS. These highly significant trends were repeated for women with insulin ≥ 10 mU/L.

**FIGURE 2:**
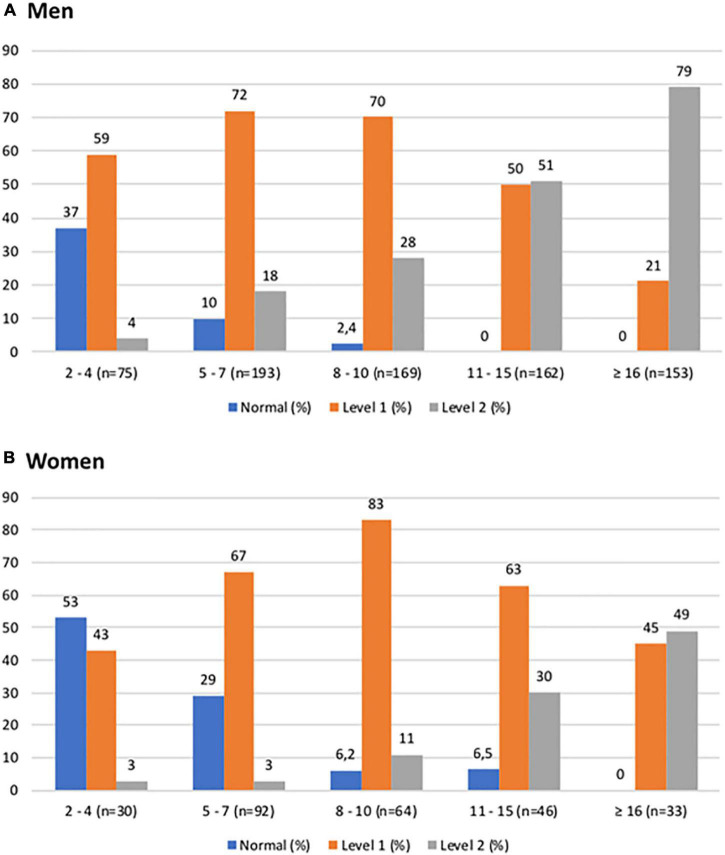
Proportion of patients classified as normal, Level 1 MetS, or Level 2 MetS, vs. ranges of fasting insulin (mU/L) for **(A)** men and **(B)** women.

The participants were then conveniently subdivided into insulin ranges from 2–4, 5–7, 8–10, 11–15 and ≥ 16 mU/L (Figure 2). This showed a lower percentage of normal individuals and a higher percentage of patients with MetS in parallel with the increase in serum insulin values.

According to our definition (Table 4), metabolically normal individuals are normotensive. The mean insulin concentrations found in both male and female participants with Level 1 MetS and Level 2 MetS were significantly higher than the insulin means of metabolically normal patients. The mean insulin of Level 2 MetS patients was also statistically higher to that of Level 1 MetS (e.g., the higher the MetS level of MetS, the higher the insulin values in men and women). These highly significant differences were also reflected in the HOMA-IR values.

**TABLE 4:** Insulin and HOMA-IR parameters in normal, Level 1 MetS, or Level 2 MetS patients, and% hepatic steatosis (HS) and hypertension (HTN) in each group.

Gender	n	Insulin (mU/L) mean ± SD (range)	HOMA-IR mean ± SD (range)	HS (%)	HTN (BP ≥ 130/85) (%)
**Men**					
Normal	49	4.6 ± 1.8 (2–10)	1.01 ± 0.43 (0.4–2.4)	4	0
Level 1 MetS	428	9.3 ± 5.5^a^* (2–48)	2.1 ± 1.33^c^* (0.4–11.6)	31	40
Level 2 MetS	265	15.6 ± 9.0^a^* (2–60)	3.9 ± 2.47^c^* (0.9–18)	60	83
**Women**					
Normal	48	5.6 ± 2.3 (2–13)	1.20 ± 0.50 (0.4–3)	0	0
Level 1 MetS	168	9.1 ± 4.9^b^* (3–34)	2.00 ± 1.65^d^* (1.6–7.9)	13	21
Level 2 MetS	39	15.4 ± 7.1^b^* (5–31)	3.9 ± 2.03^d^* (1.1–7.9)	49	78

HS, Hepatic Steatosis; HTN, Hypertension; a-d: comparison among groups according to the respective letter; 752 men (1 patient’s insulin was not measured); 265 women; 1,017 total.

*Significantly different to normal—the effects were large, so *p* values were < 0.001.

Similar trends for insulin and HOMA-IR were also observed for the proportions of hepatic steatosis (HS) in both men and women. However, 10% of men with two normal anthropometric parameters (and 4% of men with no IDF criteria) were found to have hepatic steatosis, possibly related to alcohol intake or another cause. The proportion of patients with hypertension was significantly greater among men with Level 2 MetS compared to men with Level 1 MetS, and this trend was also observed among the women.

As metabolic syndrome may progress to renal failure and albuminuria, renal function was also assessed in the patient cohort by serum creatinine and microalbuminuria. However, no significant differences were found between the normal and MetS groups. This may have been the result of the late evolution of renal complications.

## Discussion

The prevalence of metabolic syndrome (MetS) varies between countries and between regions of the same country. In a study conducted in a city in the southern region of Brazil (Paraná State), MetS was confirmed in approximately 50% of the adult population over 40 years of age and in both genders (25). In another study carried out in the city of Niterói (Southeast Region, Brazil), a prevalence of about 60% was observed in older adults, based on the criteria defined by the IDF (26).

MetS is a set of clinical abnormalities which include obesity, hypertension, dyslipidemia, hyperglycemia, and hyperinsulinemia. It is associated with a prolonged subclinical inflammatory process evidenced by a series of biomarkers such as pro-inflammatory cytokines, pro-oxidants, and prothrombotic factors (1, 4, 27–31).

Hyperinsulinemia has long been considered secondary to insulin resistance resulting from an inflammatory process generated by adiposity. However, it has more recently been found to be directly responsible for the inflammatory condition and for obesity (32–37). Insulin has a double action in the endothelium. It normally plays a protective role by increasing the production of nitric oxide, which is an important vasodilator and anti-aggregant that limits the growth of muscle cells. Insulin can also interfere with the release of endothelin ET-1, which is a potent vasoconstrictor. However, the beneficial effects of insulin that predominate under normal conditions are reversed in the face of insulin resistance and hyperinsulinemia (29).

Hyperinsulinemia increases the apoptosis of endothelial progenitor cells that are important for the maintenance of endothelial function and that may promote both muscle cell proliferation and atherogenesis (29, 30, 38, 39). It can also cause a specific form of cardiomyopathy, which is characterized by diastolic dysfunction, fibrosis, and heart failure, regardless of hypertension and atherosclerosis. This is secondary to abnormal coronary microcirculation, and activation of the sympathetic nervous and renin-angiotensin-aldosterone systems (17). It should be noted that hyperinsulinemia is a risk factor regardless of whether the patient is thin or obese, (40) diabetic or not (41).

The presence of hyperinsulinemia is clearly responsible for MetS damage, yet despite this importance, the reference points for insulin levels are variable and often incorrect. Some clinical analysis laboratories have established normal insulin limits up to 25 mU/L, or more. Other authors (37, 42) have defined fasting hyperinsulinemia as insulin levels of above 12 mU/L, while still others have suggested limits below 10 mU/L (43). Hyperinsulinemia has also been defined as the 75th percentile of the sum of the distribution in the normotensive, non-obese group, with normal glycemia (44).

The cut-offs obtained for the diagnosis of hyperinsulinemia (and raised HOMA-IR) in the present study [i.e., Men ≥ 8 mU/L (≥ 1.5), Women ≥ 10 mU/L (≥ 2.0)], are lower than those considered as the reference points by most clinical analysis laboratories. As can be observed, only 8% of men and 6% of women with insulin above the cut-offs defined for the diagnosis of hyperinsulinemia were considered metabolically normal in our cohort.

The proportion of participants considered metabolically normal significantly decreased with insulin values above the cut-offs and increased in participants with Level 1 or Level 2 MetS. This distribution clearly differentiated between the groups, validating the cut-off defined for the diagnosis of hyperinsulinemia, and consolidating the importance of the metabolic classes proposed in this analysis (normal, Level 1, and Level 2 MetS). These data were even more evident in Figure 2, which expanded the hyperinsulinemia ranges.

The various criteria used for diagnosis of MetS and its associated complications certainly do not appear concurrently. Instead, they appear over time and are preceded by prolonged exposure to hyperinsulinemia, which can be an earlier marker of metabolic risk (21–24, 30, 31, 43, 45–49).

A clear definition of the diagnostic cut-off for hyperinsulinemia will allow for early prevention and therapeutic intervention, before the emergence of the recognized and multiple morbidities associated with MetS, such as type 2 diabetes, cardiovascular diseases, and neoplasms. Our proposal to classify individuals as metabolically normal, Level 1 MetS, or Level 2 MetS is aimed at recognizing the clinical importance of hyperinsulinemia and the early stages of the syndrome, regardless of the number of IDF criteria exhibited by a patient.

Insulin values were significantly higher in patients with Level 1 MetS than in those considered metabolically normal (i.e., without any of the five IDF criteria defined previously), as was the prevalence of steatosis and hypertension. This showed that the presence of one or two of the IDF criteria already suggests the presence of insulin resistance and metabolic dysfunction. Without effective medical intervention, these individuals will likely develop MetS to its fullest extent over time, as evidenced by participants with Level 2 MetS who had even greater hyperinsulinemia and/or HOMA-IR values, and a higher prevalence of HTN and HS, compared to patients with Level 1 MetS. No significant changes in renal function, as assessed by the means for serum creatinine or microalbuminuria, were detected between the normal vs. the MetS groups, probably because these are late complications.

Our study has some limitations. First, it is a retrospective study with data from Brazilian patients who underwent annual check-up exams, which makes the study findings valid only for Brazil. However, such design of study can serve as a model for other countries and, perhaps, contribute to standardization of insulin reference levels at a global level. In addition, there is no information about the dietary and physical activity patterns of these individuals what may contribute to the MetS.

In summary, studies to evaluate the benefits of an early approach of patients with fasting insulin values above normal cut-offs, i.e., 8 mU/L in men and 10 mU/L in women (and/or HOMA-IR upper limits of 1.5 and 2.0), despite the number of diagnostic criteria for MetS (Level 1 MetS) to reduce cardiovascular events are necessary.

## Data availability statement

The original contributions presented in this study are included in the article/supplementary material, further inquiries can be directed to the corresponding author.

## Ethics statement

This study was approved by the ethics and research committee of Hospital Alemão Oswaldo Cruz de São Paulo (CAAE: 46489021.6.0000.0070). Written informed consent for participation was not required for this study in accordance with the national legislation and the institutional requirements.

## Author contributions

PC: conception and design, data acquisition, analysis and interpretation, drafting the article, critically revising the article, funding acquisition, and supervision of the research group. JD: data interpretation, drafting the article, and critically revising the article. VS, PM, and AC-N: data analysis and interpretation, drafting the article, and critically revising the article. SM, ÉO, LP, AB, and FS: data analysis and interpretation and critically revising the article. All authors have read and approved the final version of the manuscript.

## Abbreviations

BMI: Body mass index
HDL-C: high density lipoprotein cholesterol
HS: hepatic steatosis
HOMA-IR: Homeostatic Model Assessment for Insulin Resistance
HTN: Hypertension
IDF: International Diabetes Federation
MetS: Metabolic Syndrome.
